# Overexpression of JAM-A in Non-Small Cell Lung Cancer Correlates with Tumor Progression

**DOI:** 10.1371/journal.pone.0079173

**Published:** 2013-11-12

**Authors:** Min Zhang, Wenting Luo, Bo Huang, Zihui Liu, Limei Sun, Qingfu Zhang, Xueshan Qiu, Ke Xu, Enhua Wang

**Affiliations:** 1 Department of Pathology, First Affiliated Hospital and College of Basic Medical Sciences, China Medical University, Shenyang, People’s Republic of China; 2 Department of Pathology, College of Basic Medical Sciences, Shenyang Medical College, Shenyang, People’s Republic of China; 3 Department of Pathology, Liaoning Cancer Hospital, Shenyang, People’s Republic of China; 4 Department of radiology, First Affiliated Hospital of China Medical University, Shenyang, People’s Republic of China; H. Lee Moffitt Cancer Center & Research Institute, United States of America

## Abstract

The objective of the current study was to determine the clinical significance of junctional adhesion molecule A (JAM-A) in patients with non-small cell lung cancer (NSCLC) and the biological function of JAM-A in NSCLC cell lines. We showed that JAM-A is predominantly expressed in cell membranes and high expression of JAM-A occurred in 37% of lung tumor specimens compared to corresponding normal tissues. High expression of JAM-A was significantly correlated with TNM stage (P = 0.021), lymph node metastasis (P = 0.007), and decreased overall survival (P = 0.02), In addition, we observed that silencing JAM-A by small interfering RNA inhibited tumor cell proliferation and induced cell cycle arrest at the G1/S boundary. Western blotting analysis revealed that knockdown of JAM-A decreased the protein levels of cyclin D1, CDK4, 6, and P-Rb. Thus, JAM-A plays an important role in NSCLC progression.

## Introduction

Junctional adhesion molecule A (JAM-A) is a type I transmembrane glycoprotein that belongs to the immunoglobulin superfamily. JAM-A is widely distributed in tissues, including placenta, lungs, liver, kidneys, pancreas, heart, brain, intestines, and lymph nodes [1], [2], [3], [4]. JAM-A is expressed predominantly in intercellular junctions of epithelial and endothelial cells, JAM-A is also found on the surface of leukocytes, lymphocytes, platelets, and erythrocytes [5], [6], [7], [8]. JAM-A is implicated in diverse cellular processes, such as cell-cell adhesion, leukocyte migration, platelet activation, angiogenesis, and reovirus binding [5], [9], [10], [11]. The JAM-A protein is composed of an extracellular domain with two Ig-like loops, a single membrane-spanning region, and a short cytoplasmic tail terminating in a PDZ-binding motif. JAM-A can form homodimers through this N-terminal Ig loop. Homophilic interactions are important for the aforementioned JAM-A function in cells [12]. The C-terminal PDZ-binding motif can facilitate interactions with various scaffold proteins, such as ZO-1, AF-6, and PAR-3 [13], [14], [15]. JAM-A dimerization and PDZ binding motifs are critical for the signaling cascade [16], [17]. Recently, JAM-A has been implicated in tumor progression, but the role of JAM-A in tumor growth and dissemination remains a controversial issue. Naik et al. [18] initially reported that JAM-A could reduce invasion and motility of breast cancer cell lines *in vitro* and JAM-A expression in breast cancer patients was negatively associated with tumor aggressiveness and metastasis. Similar clinical- or *in vitro*-generated data were reported by others involving endometrial carcinoma [19] and pancreatic cancer [20]. On the contrary, Mc Sherry et al. [21], using a larger clinical data set, reported a positive correlation between JAM-A expression and invasive breast cancer prognosis. Similarly, larger clinical-, *in vitro*-, and *in vivo*-generated data sets were recently reported in breast cancer and epidermoid carcinoma [22], [23], [24], [25], [26]. The expression of JAM-A protein in non-small cell lung cancer (NSCLC) tissues and the relationship with various clinicopathologic factors has not been completely investigated. Furthermore, the exact role of JAM-A in lung cancer progression is unclear. Therefore, we determined JAM-A expression in NSCLC tissues by immunohistochemistry. In addition, we determined the association between JAM-A expression and proliferation in several NSCLC cell lines. We showed that JAM-A is expressed relatively high amounts in NSCLC tissues and some types of NSCLC cell lines, and that cell membrane-associated JAM-A levels are correlated with tumor aggressiveness.

## Materials and Methods

### Ethics Statement

This study was conducted with the approval of the Institutional Review Board of the China Medical University. Written informed consent was obtained from all NSCLC patients and all clinical investigations were conducted according to the principles promulgated in the Declaration of Helsinki.

### Patients and Specimens

Eighty-two NSCLC samples were obtained from the First Affiliated Hospital of China Medical University between 2002 and 2009; 42 samples underwent follow-up studies. None of the patients received radiotherapy or chemotherapy before surgical resection. The histologic diagnosis and grade of differentiation of the tumors were defined by evaluation of hematoxylin and eosin-stained tissue sections according to the World Health Organization guidelines of classification. All 82 specimens were re-evaluated with respect to histologic subtype, differentiation status, and tumor stage. Squamous cell carcinoma and adenocarcinoma were identified in 36 and 46 of the 82 cases, respectively. Lymph node metastases were observed in 31 patients. Tumors were classified into stages I (n = 45), II (n = 30), and III (n = 17) according to the p-TNM staging system of the International Union Against Cancer (7th edition). Overall survival was defined as the period of time from initial diagnosis to death or the date of last follow-up.

### Cell Lines and Cell Culture Conditions

HBE, A549, H1299, H157, and H460 cell lines were obtained from the American Type Culture Collection (Manassas, VA, USA). SPC and LK2 cell lines were purchased from the Shanghai Cell Bank of Chinese Academy of Science. The cells were cultured in RPMI-1640 (Invitrogen, Carlsbad, CA, USA) containing 10% fetal calf serum (FBS; Invitrogen), 100 IU/ml of penicillin (Sigma, St. Louis, MO, USA), and 100 mg/ml of streptomycin (Sigma). Cells were grown on sterile tissue culture dishes and passaged every 2 days using 0.25% trypsin (Invitrogen).

### Immunohistochemistry Analysis

Surgically-excised tumor specimens were fixed with 10% neutral formalin, embedded in paraffin, and 4-µm thick sections were prepared. Immunostaining was performed using the ElivisionTM plus Polyer HRP (mouse/rabbit) IHC Kit (Maixin, Fuzhou, China). The sections were deparaffinized in xylene, rehydrated in graded alcohol series, and boiled in 0.01 M EDTA buffer (pH 9.0) for 20 minutes in a boiler. Endogenous peroxidase activity was blocked using hydrogen peroxide (0.3%), which was followed by incubation with normal goat serum to reduce non-specific binding. Tissue sections were incubated with JAM-A rabbit monoclonal antibody (1∶100 dilution; Abcam, Cambridge, MA, USA). Rabbit immunoglobulin, at the same concentration as the antigen-specific antibody (Maixin) was used as a negative control. Staining for all primary antibodies was performed at 4°C overnight. ElivisionTM plus Polyer HRP was used as the secondary antibody. After washing, the sections were incubated with 3, 3′-diaminobenzidine tetrahydrochloride to develop the peroxidase reaction. Counterstaining of the sections was done with hematoxylin, which were then dehydrated in ethanol before mounting. Two independent investigators examined all tumor slides randomly. Five views were examined per slide, and 100 cells were observed per view at 400X magnification. Immunostaining of JAM-A was scored following a semi-quantitative scale by evaluating representative tumor areas; the intensity and percentage of cell membranes had higher immunostaining than the control cells. Membrane staining of the tumor cells was considered to be positive immunostaining. The intensity of JAM-A membranes staining was also scored as 0 (no staining), 1 (weak), 2 (moderate), and 3 (high). Percentage scores were assigned as follows: 1, 1%–10%; 2, 11%–50%; 3, 51%–80%; and 4, 81%–100% [20]. The scores of each tumor sample were multiplied to give a final score of 0–12 and the total expression of JAM-A was determined as low (≤4) or high expression (>4).

### Quantitative Real-time PCR (SYBR Green Method)

Quantitative real-time PCR was performed using SYBR Green PCR master mix (Applied Biosystems, Foster City, USA) in a total volume of 20 ul on a 7900HT Fast Real-Time PCR System (Applied Biosystems) as follows: 95°C for 30 seconds; 40 cycles at 95°C for 5 seconds; and 60°C for 30 seconds. A dissociation step was performed to generate a melting curve to confirm the specificity of the amplification. β-actin was used as the reference gene. The relative levels of gene expression were represented as ΔCt = Ct gene–Ct reference and the fold change of gene expression were calculated by the 2^−ΔΔCt^ method, where ΔΔCt = (Ct _gene_–Ct _reference_) _sample_– (Ct_ gene_–Ct _reference_) _calibrator_. Primer sequences of JAM-A were 5′-GTG AAG TTG TCC TGT GCC TAC TC-3′ (forward) and 5′-ACC AGT TGG CAA GAA GGT CAC C-3′ (reverse). Primer sequences of β-actin were 5′- TGC CTA GAT CAA GAA GCA G -3′ (forward) and 5′- TGC CTA GAT CAA GAA GCA G -3′ (reverse). Three independent experiments were conducted in triplicate under identical conditions.

### Small Interfering RNA Treatment

Small Interfering RNA (siRNA) for JAM-A was synthesized by Genepharma (Shanghai, China). The sequences of siRNA were as follows: 5′- GAA GUG AAG GAG AAU UCA ATT-3′ (sense); and 5′-UUG AAU UCU CCU UCA CUU CTT-3′ (antisense). The sequences of non-targeting siRNA (negative control) used as a negative control were as follows: 5′-UUC UCC GAA CGU GUC ACG UTT-3′ (sense); and 5′-ACG UGA CAC GUU CGG AGA ATT-3′ (antisense). Cells were seeded in a 6-well plate 24 h before transfection experiments. The cells were transfected with 100 pmol JAM-A siRNA or negative control siRNA using Lipofectamine2000 (5 ul/well; Life Technologies Corporation, Grand Island, NY, USA) according to the manufacturer’s protocol. Following transfection, the protein and mRNA levels of JAM-A were assessed 48 h later. Three independent experiments were conducted under identical conditions.

### Western Blot Analysis

Total protein from cells was extracted in lysis buffer (50 mM Tris-HCl [pH 8.0], 150 mM NaCl, 0.5% Nonidet P40, 0.5% sodium deoxycholate, and phenylmethylsulfonyl fluoride [PMSF]; Beyotime, Haimen, China) and quantified using the bicinchoninic acid (BCA) method (Beyotime). Sixty µg of protein was separated by SDS–PAGE (10%) and transferred to polyvinylidene fluoride (PVDF) membranes (Millipore, Billerica, MA, USA), After blocking with 5% BSA in Tris-buffered saline-Tween 20 (TBST; 20 mM Tris–HCl, 500 mM NaCl, and 0.05% Tween-20), membranes were incubated at 4°C overnight with the following primary antibodies: JAM-A (1∶1000; Abcam); and anti-P-Rb, anti-P27, anti-P21, anti-cyclin A, anti-cyclin B, anti-cyclin D1, anti-CDK4, anti-CDK6,anti-AKT1/2, and anti-P-AKT Thr 308 (1∶1000; Cell Signaling Technology, Danvers, MA). After incubation with peroxidase-coupled anti-mouse or rabbit IgG (Santa Cruz Biotechnology, Inc., Santa Cruz, CA, USA) at 37°C for 2 h, bound proteins were visualized using ECL (Thermo Fisher Scientific, Waltham, MA, USA) and detected using BioImaging Systems (UVP Inc., Upland, CA, USA). The relative protein levels were calculated based on GAPDH as the loading control. Three independent experiments were conducted under identical conditions.

### Flow Cytometry Assay

Cells were trypsinized and 1×10^6^ cells were washed twice with chilled incubation buffer (2% BSA in PBS) and centrifuged, then the cells were resuspended in 100 uL of incubation buffer and incubated with or without FITC-conjugated mouse anti-human JAM-A antibody (1∶100; Biolegend, San Diego, CA, USA) or FITC-conjugated mouse IgG1, κ isotype control antibody (1∶100; Biolegend) on ice for 30 min in the dark. The cells were then washed twice with incubation buffer and processed for flow cytometric analysis. Data were analyzed using FlowJo 7.6.1 software. Three independent experiments were conducted in triplicate under identical conditions.

### Cell Proliferation Test

A cell proliferation assay was performed using MTT (Sigma) according to the manufacturer’s protocol. Briefly, H1299 and A549 cells were transiently transected with JAM-A siRNA or negative control siRNA. After 24 h, the cells were trypsinized and seeded at a concentration of 3×10^3^ cells/100 ul/well in 96-well culture plates overnight. Each subsequent day, cells were treated with 20 ul (5 mg/ml)/well of MTT solution during the last 4 h of the culture. The culture media was replaced with DMSO (150 ul/well). The optical density of the wells was measured at 490 nm using a microplate reader. Three independent experiments with 5 internal replicates per experiment were conducted under identical conditions.

### Colony Formation Assay

For colony formation assays, cells were transfected with JAM-A or negative control siRNA for 48 h, then plated into 6-well culture plates (1000 per well) and incubated for 12 days. Plates were washed with PBS and stained with Giemsa. The number of colonies with >50 cells was counted. The colonies were manually counted using a microscope. Three independent experiments were conducted in triplicate under identical conditions.

### Cell Cycle Analysis

Cells (100,000) were seeded into 6-well culture plates, synchronized after serum starvation for 24 h, then transfected with the indicated amounts of siRNA. Cells were harvested, fixed in 75% ethanol 48 h after transfection, washed with PBS, and stained in 5 mg/ml of propidium iodide in PBS supplemented with RNase A (Roche, Indianapolis, IN, USA) for 30 min at room temperature. Data were collected using BD systems. Three independent experiments were conducted under identical conditions.

### Statistical Analysis

SPSS (version 16.0 for Windows; SPSS, Inc., Chicago, IL, USA) was used for all analyses. The chi-squared test was used to determine the correlation between JAM-A expression and clinicopathologic characteristics. Kaplan–Meier curves were used for survival analysis, and log-rank was determined based on the differences. Student’s t-test was used to compare other data. P-values were based on two-sided statistical analysis, and a p<0.05 was considered to indicate statistical significance.

## Results

### Expression and Distribution of JAM-A in NSCLC Tissues

**Figure 1:**
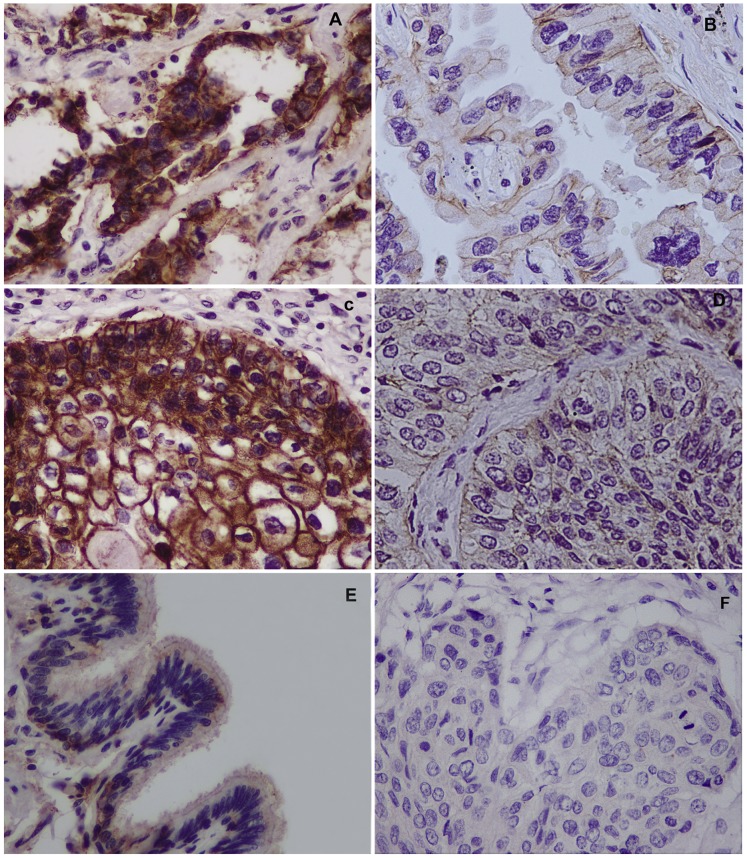
Immunohistochemical staining of JAM-A in lung cancer tissue sections. (A) Strong JAM-A expression in lung adenocarcinoma (400X). (B) Weak JAM-A expression in lung adenocarcinoma (400X). (C) Strong JAM-A expression in lung squamous cell carcinoma (400X). (D) Weak JAM-A expression in lung squamous cell carcinoma (400X). (E) Weak staining in normal bronchial epithelium in non-cancerous lung tissue (400X). (F) Negative controls for JAM-A staining with non-immune rabbit IgG antibody (400X).

**Figure 2:**
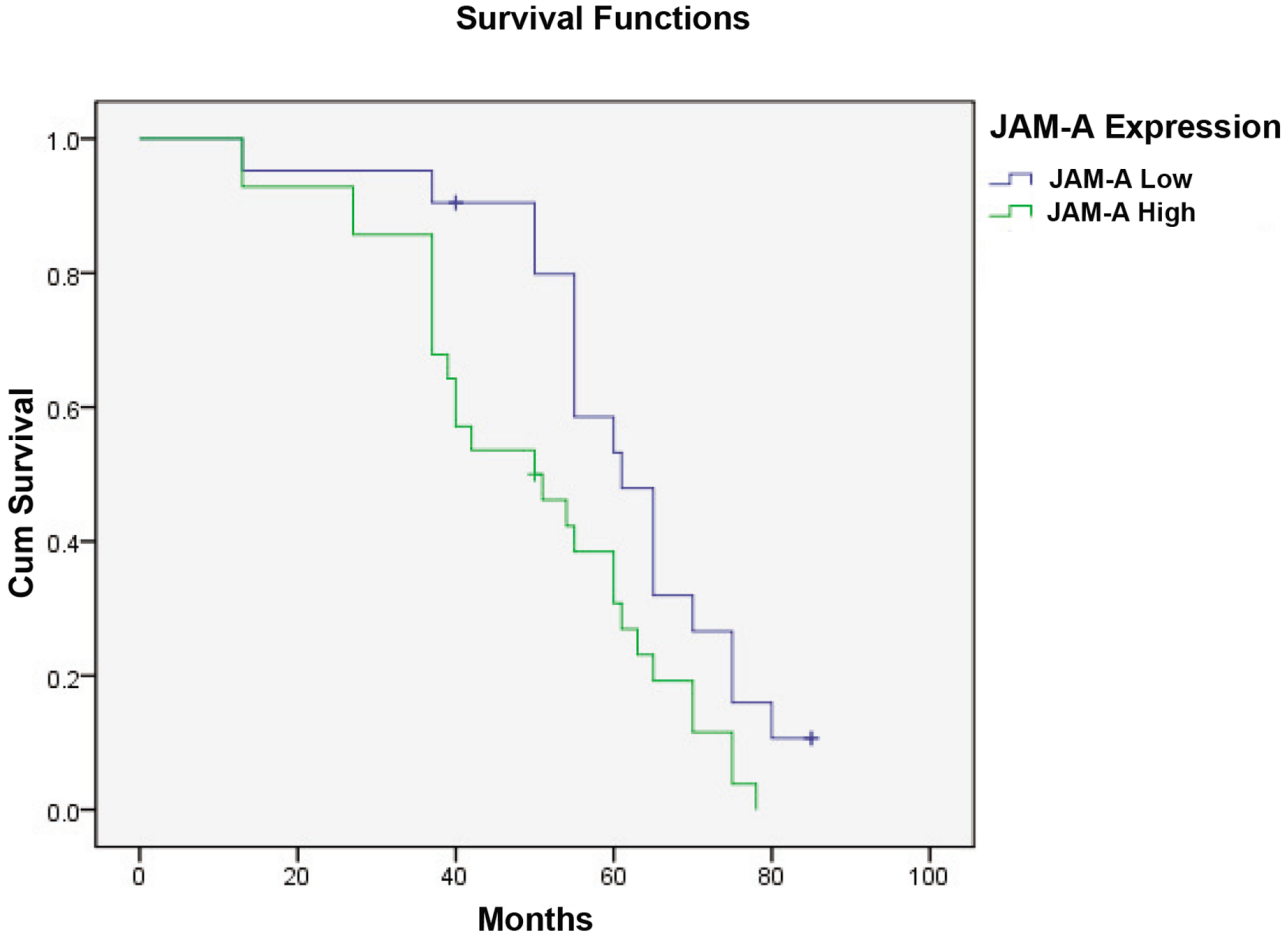
Kaplan–Meier survival curves of NSCLC patients. Patients with low expression of JAM-A had better overall survival than did patients with high expression of JAM-A, as defined by the log-rank test (P = 0.02).

We analyzed the expression and distribution of JAM-A in 82 NSCLC specimens and the corresponding normal tissues using immunohistochemistry. JAM-A was predominantly expressed in cell membranes; high expression of JAM-A was found in 37% of lung tumor specimens (Table 1), which was much higher than bronchial epithelia and pneumocytes in corresponding non-tumorous lung tissues (Fig. 1). We investigated the relationship between the expression of membrane JAM-A and clinicopathologic parameters. Based on univariate analysis, high expression of membrane JAM-A was significantly correlated with TNM stage (P = 0.021) and lymph node metastases (P = 0.007), but not with age (P = 0.818), gender (P = 0.96), differentiation (P = 0.174), tumor status (P = 0.105), and tumor histology (P = 0.818; Table 1). We evaluated the relationship between the expression of membrane JAM-A and overall survival in 49 NSCLC patients. After Kaplan-Meier survival analysis, we found that patients with high expression of membrane JAM-A had a significantly lower survival (median survival = 50±8.92 months; 95% confidence interval [CI], 32.56–67.48 months) than patients with low expression of membrane JAM-A (median survival = 61±4.31 months; 95% CI, 52.56–69.45 months, P = 0.02; Fig. 2).

**Table 1:** Distribution of JAM-A status in NSCLC according to clinicopathologic characteristics.

Characteristics	Patients	F11R low expression	F11R high expression	p
Age (years)				
<60	35	23(65.7)	12(34.3)	0.818
≧60	47	29(61.7)	18(38.3)	
Gender				
Male	38	24(63.2)	14(36.8)	0.96
Female	44	28(63.6)	16(36.4)	
Histology				
Adenocarcinoma	46	30(65.2)	16(34.8)	0.818
Squamous cell carcinoma	36	22(61.1)	14(38.9)	
Differentiation				0.174
Well	38	21(55.3)	17(44.7)	
Poor-moderate	44	31(70.5)	13(29.5)	
TNM stage				0.012
I	45	34(77.3)	11(22.7)	
II–III	37	18(48.6)	19(51.4)	
Tumor status				0.105
T1	35	26(74.3)	9(25.7)	
T2–T4	47	26(55.3)	21(44.7)	
Nodal status				0.007
N0	51	38(74.5)	13(25.5)	
N1–N3	31	14(45.2)	17(54.8)	

### JAM-A Depletion Inhibits Lung Cancer Cell Proliferation

**Figure 3:**
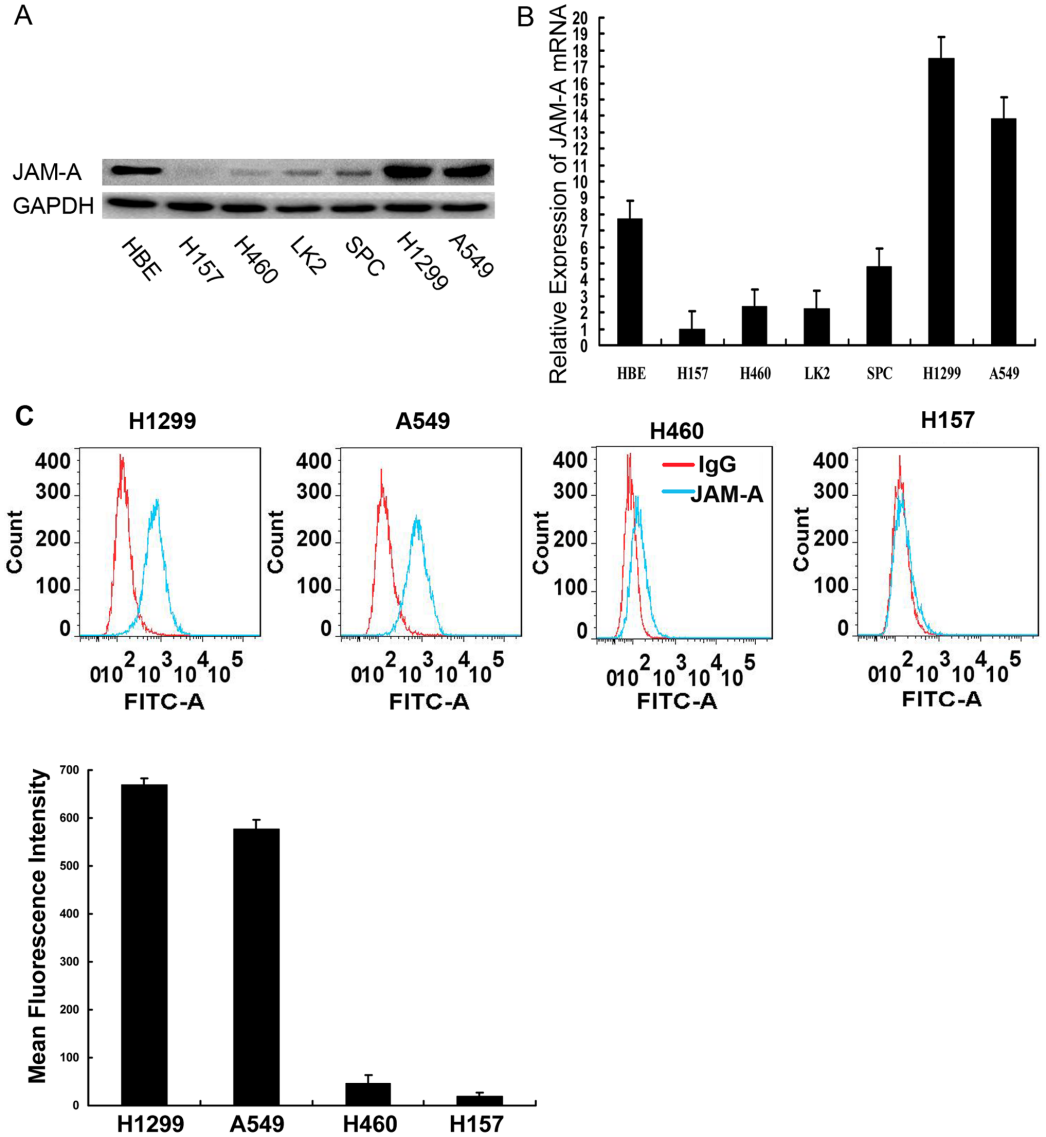
JAM-A expression in NSCLC cells. JAM-A expression in a panel of human airway-derived cell lines assessed by Western blot, Data are shown as representative Western blots experiments. Experiments were independently repeated 3 times with similar results. (B) Relative expression of JAM-A mRNA in a panel of human airway-derived cell lines assessed by real-time PCR. The relative quantity of JAM-A mRNA, normalized to β-actin, were compared to H157 cells based on the equation RQ = 2^−ΔΔCt^. Columns, mean of RQ of triplicate values in a representative experiment; bars, max/min RQ. Experiments were independently repeated 3 times and conducted in triplicate with similar results. (C) Surface expression of JAM-A in a panel of human airway-derived cell lines assessed by a flow cytometry assay. Confluent cells were trypsinized and flow cytometry assay analyses were conducted as described in the Methods section. Data are shown as representative histograms (top panel), mean fluorescence intensity of triplicate values in a representative experiment, Columns, mean; bars,SD. (bottom panel). Experiments were independently repeated 3 times in triplicate with similar results.

We detected JAM-A expression in normal bronchial epithelial (HBE) cells and six lung cancer cell lines by Western blot and real-time PCR analysis. H1299 and A549 cells exhibited high levels of expression of JAM-A, HBE cells exhibited moderate levels of expression of JAM-A, and H157, H460, SPC, and LK2 exhibited low levels of expression of JAM-A (Fig. 3A, B). Because JAM-A is a type I transmembrane glycoprotein, we then analyzed cell membrane-associated JAM-A levels using a flow cytometry assay in relatively high endogenous JAM-A cell lines (H1299 and A549) and relatively low endogenous JAM-A cell lines (H460 and H157). As shown in Fig. 3C, expression of surface JAM-A in H1299 and A549 cells was much higher than H157 and H460 cells. Thus, we used H1299 and A549 cells in loss-of-function studies.

**Figure 4:**
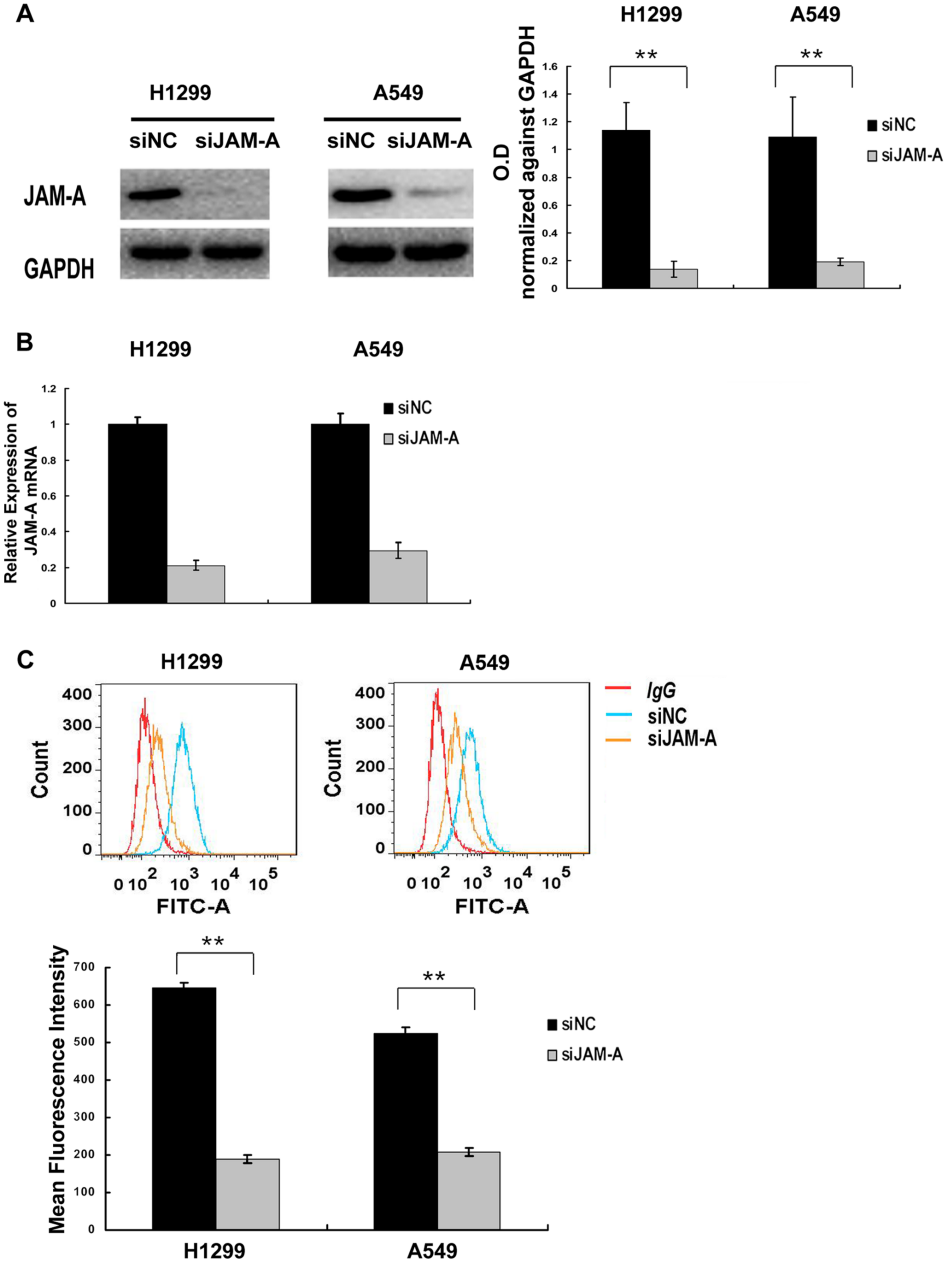
Suppression of JAM-A expression in H1299 and A549 cells. (A) Western blot analyses of JAM-A depletion efficiency in cancer cells. Data are shown as representative Western blots (left panel). Densitometry value analysis of 3 independent Western blot experiments, data were normalized against GAPDH, Columns, mean; bars, SD, **p<0.01(right panel). (B) Real-time PCR analyses of JAM-A depletion efficiency in cancer cells, The relative quantity of JAM-A mRNA, normalized to β-actin, were compared to the negative control siRNA-transfected group based on the equation RQ = 2^−ΔΔCt^. Columns, mean of RQ in a representative experiment; bars, max/min RQ. Experiments were independently repeated 3 times in triplicate with similar results. (C) Flow cytometry assay analyses of JAM-A surface expression depletion efficiency. H1299 and A549 cells were transiently transected with negative control siRNA or JAM-A siRNA; after 48 h, cells were trypsinized and flow cytometry assay analyses were conducted as described in the Methods section. Data are shown as representative histograms (upper panel), mean fluorescence intensity of triplicate values in a representative experiment, Columns, mean; bars, SD. **p<0.01 (lower panel). Experiments were independently repeated 3 times in triplicate with similar results.

**Figure 5:**
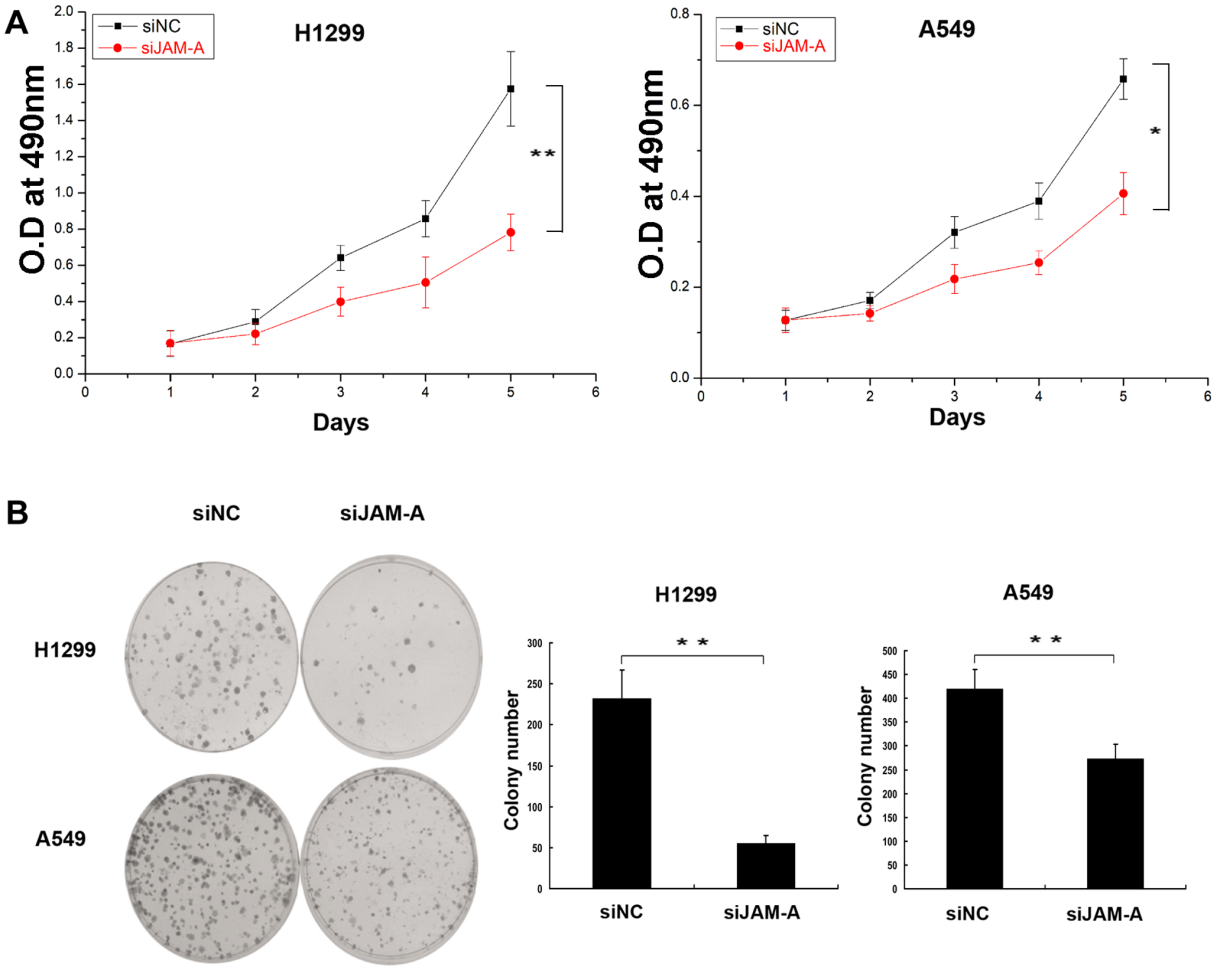
JAM-A knockdown inhibited cancer cell proliferation. (A) MTT assay was performed after JAM-A siRNA treatment. The absorbance at 490 nm at different time points was observed. Data are shown as the mean ± SD of representative experiments, *p<0.05; **p<0.01. 3 independent experiments with 5 internal replicates per experiment were performed with similar results. (B) Assessment of clonogenic potentials of the JAM-A-depleted cancer cells. Complete fields per plate were photographed and presented. Data are shown as representative colony formation assay (Left panel), the number of colonies of triplicate values in a representative experiment was counted, Columns, mean; bars, SD. **p<0.01 (Right panel). 3 independent experiments in triplicate were conducted with similar results.

To determine whether or not JAM-A takes part in modulating lung cancer cell growth, we used siRNA to knockdown JAM-A expression in H1299 and A549 cell lines. Compared with negative control siRNA-transfected cell lines, JAM-A-specific siRNA effectively suppressed total JAM-A protein levels (Fig. 4A), JAM-A mRNA levels (Fig. 4B), and surface expression levels (Fig. 4C) in the two tested cell lines. The cell growth rate was determined by MTT assay. H1299 and A549 cells transfected with JAM-A-specific siRNA had reduced growth rates compared to negative control siRNA (Fig. 5A). We then conducted an independent method (colony formation assay) to validate the anti-proliferative effect of JAM-A inhibition in lung cancer cells. In agreement with the MTT results, JAM-A knockdown effectively reduced the capacity of colony formation of the 2 tested lung cancer cell lines (negative control vs. JAM-A siRNA, H1229∶231±19 vs. 55±7, p<0.01; A549∶420±31 vs. 273±21, p<0.01; Fig. 5B).

### JAM-A Knockdown Results in G1 Arrest and Growth Inhibition of Lung Cancer Cells

**Figure 6:**
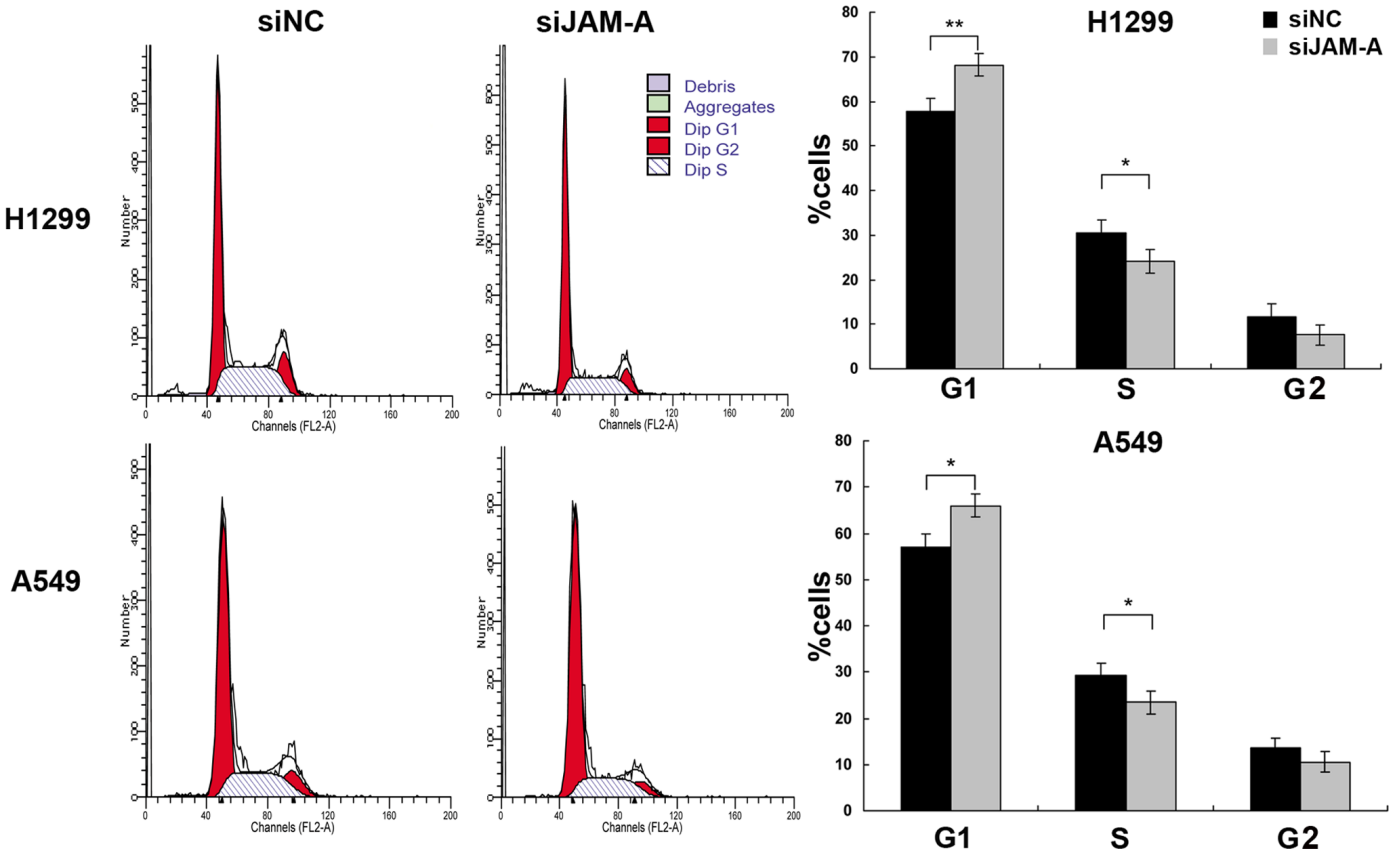
JAM-A knockdown impaired cell cycle progression. Effectiveness of JAM-A knockdown on cell cycle progression was analyzed by fluorescence-activated cell sorting. Data are shown as a representative experiment (Left panel), the percentage of cells in different cell cycle phase is shown as the mean ± SD of 3 independent experiments, *p<0.05; **p<0.01 (right panel).

**Figure 7:**
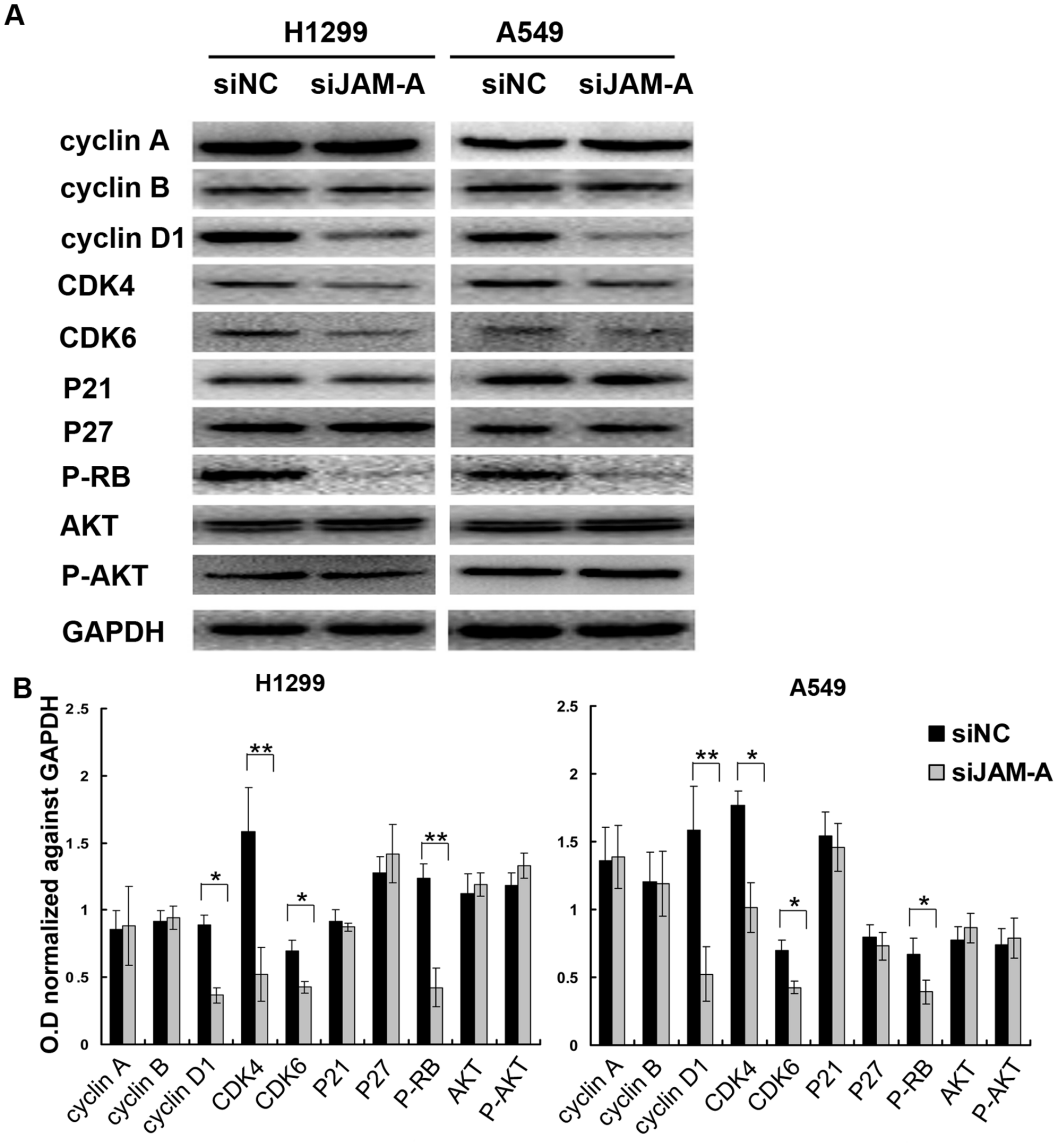
Expression of a panel of proteins in JAM-A-depleted lung cancer cells. (A) Western blot analysis of a panel of proteins was conducted on siNC- or siJAM-A-transfected H1299 cell lines (left panel) and A549 cell lines (right panel). Data are shown as representative Western blots. Three independent experiments were conducted with similar results. (B) Densitometry analysis of 3 independent Western blot experiments, data were normalized against GAPDH, Columns, mean; bars, SD, *p<0.05; **p<0.01.

We next sought to investigate possible mechanisms whereby JAM-A knockdown can decrease cell proliferation. We analyzed the cell cycle by fluorescence-activated cell sorting (FACS), and showed that the percentage of cells in the G1 phase was increased (H1299, negative control vs. JAM-A siRNA, 57.8±2.8 vs. 68.1±2.5, p<0.01; A549 negative control vs. JAM-A siRNA, 57.12±2.77 vs. 65.96.1±2.52, p<0.05; Fig. 6), whereas cells in the S phase were decreased in cells treated with JAM-A knockdown compared to control cells (H1299, negative control vs. JAM-A siRNA, 30.49±2.8 vs. 24.1±2.74, p<0.05; A549 negative control vs. JAM-A siRNA, 29.33±2.61 vs. 23.49.1±2.49, p<0.05; Fig. 6). These results suggest that JAM-A deletion arrests cell cycle progression at the G1/S boundary. To elucidate the mechanism underlying JAM-A depletion on cell cycle arrest, we further tested the effect of JAM-A knockdown on the level of expression of several cell cycle-related factors, which include cyclin A, cyclin B, cyclin D1, CDK4, CDK6, P27, P21, and P-Rb. Western blot analysis revealed that knockdown of JAM-A decreased the protein levels of cyclin D1, CDK4, CDK6, and P-Rb (Fig. 7). Taken together, these results indicate that inhibition of JAM-A expression induces cell cycle arrest in the G1 phase and suppresses lung cancer cell growth.

### PI3K-Akt-β-catenin Signal Cascade is not Inhibited by High Levels of Expression of Surface JAM-A in Lung Cancer Cells

Published studies indicate that JAM-A restricts intestinal epithelial cell (IEC) proliferation by inhibiting PI3K-Akt-β-catenin signal cascade activation [27]. To explore whether or not this signal pathway is also effective in lung cancer cell lines, we also tested the total and phosphorylated levels of Akt in JAM-A loss-of-function studies. Western blot analysis revealed that knockdown of JAM-A did not change the active levels of Akt, which suggests that high JAM-A levels in H1299 and A549 cells did not affect PI3K-Akt-β-catenin cascade activation (Fig. 7).

## Discussion

JAM-A is expressed in several kinds of tumors and is implicated in tumor progression. The expression of JAM-A protein in NSCLC tissues and the relationship with various clinicopathologic factors has not been fully examined. Furthermore, the exact role of JAM-A in lung cancer progression is unclear. In this study we examined the pattern of expression and the biological function of JAM-A in NSCLC. We showed that JAM-A is predominantly localized in cell membranes, and the expression of JAM-A in lung cancer tissues is higher than corresponding normal lung tissues. The high level of expression of membrane JAM-A was significantly associated with advanced pTNM stage, lymph node metastases, and reduced overall survival in lung cancer patients. Taken together, our results revealed that JAM-A is a potential oncogenic protein in NSCLC and might serve as a negative predictor of survival in NSCLC patients.

To further explore the potential function of JAM-A in lung cancer cells, we used siRNA to knockdown JAM-A expression in H1299 and A549 cell lines, which express relatively high levels of surface JAM-A. Proliferation and colony formation in the two cell lines was significantly suppressed after silencing JAM-A. Most of the proliferative factors influence cell growth by affecting cell cycle progression, thus we analyzed the cell cycle and showed that JAM-A knockdown cells had higher levels of G1 phase and lower S phase than control cells. Thus, JAM-A silencing inhibited the G1-to-S transition in cell cycle progression, which might elucidate the mechanism of JAM-A on lung cancer cell proliferation. To determine the potential mechanism of JAM-A on cell cycle regulation, we examined the effect of JAM-A knockdown on cell-cycle related molecules. We analyzed the expression of cyclin A, cyclin B, cyclin D1, CDK4, CDK6, P21, P27, and P-RB, and found that the levels of expression of cyclin D1, CDK4, CDK6, and P-RB were decreased after JAM-A knockdown. It has been previously demonstrated that P-RB is responsible for a major G1 checkpoint, blocking S-phase entry and cell growth by physically associating with E2F factors and blocking activation of gene expression that encodes products necessary for S-phase progression. The protein complex comprised of cyclin D1, CDK4, and CDK6 will phosphorylate P-RB and promote P-RB inactivation. Continued phosphorylation on P-RB by various CDKs leads to the release of E2F, thereby facilitating the activation of genes critical for S-phase progression [28], [29], [30]. Taken together, our results indicate that JAM-A positively controls NSCLC cell proliferation by regulating expression of the cell cycle-related molecules, such as cyclin D1, CDK4, CDK6, and P-RB.

Published studies indicate that JAM-A restricts IEC proliferation by inhibiting PI3K-Akt-β-catenin activation [27]. To explore whether or not high levels of surface JAM-A of the two tested cell lines negatively affects cell proliferation in the same way as IEC, we also tested the active (phosphorylation) levels of Akt in JAM-A loss-of-function studies. Western blot analysis revealed that knockdown of JAM-A in H1299 and A549 cells did not change the active levels of Akt, which indicates that high levels of JAM-A in H1299 and A549 cells did not affect PI3K-Akt-β-catenin signal cascade activation.

The role of JAM-A in tumor growth and dissemination remains a controversial issue. Naik et al. [18] initially reported that JAM-A expression is negatively associated with breast cancer aggressiveness and metastasis in 12 tumors and the corresponding non-neoplastic tissue, as well as 50 malignant and corresponding metastatic lymph node samples. However, Mc Sherry et al. [21], using larger clinical data sets (a 270 patient invasive breast cancer tissue microarray [TMA] and an independent data set of gene expression information from 295 patients with primary breast cancer), showed a positive correlation between JAM-A expression and invasive breast cancer prognosis, which was further confirmed by their another published report using a larger data set [24]. Recently, Murakami et al. [22], using TMA of 444 patients with invasive breast cancer, also reported a similar correlation. In the context of invasive breast cancer, taking into consideration the larger data set, the association between clinicopathologic data and survival data analyzed by McSherry et al. [21], [24] and Murakami et al. [22], high JAM-A expression should be a negative prognostic factor of patient outcome. The mechanism through which tumor cells maintain high JAM-A during tumor progression remains to be defined. Gotte et al. [25] showed that miR-145, which targets and down-regulates JAM-A, is down-regulated in breast cancer cells. These data might account for the high levels of JAM-A in this particular type of tumor [22]. However, whether or not miR-145 plays the same role in NSCLC in breast cancer need further research. It should be noted that negative, but not positive effects of JAM-A on tumor progression were also reported based on clinical data sets involving endometrial carcinoma [19] and pancreatic cancer [20], suggesting that the role of JAM-A in tumor progression may be cell type-dependent. Published data have focusing on mechanisms for JAM-A regulating tumor progression, even though controversial, would better clarify this issue. Positive [21], [22], [23], [24], [25], [26] and negative [18], [19] effects of JAM-A on tumor progression have been reported *in vivo*, *in vitro,* or using a genetic mammary tumor model. Comparing all of the aforementioned studies, we speculate that JAM-A function is context-dependent. For example, Naik et al. [18] reported that JAM-A reduces invasion and motility of MDA-MB-231 breast cancer cells, but Gotte et al. [25], using the same type of cells, showed a contradictory role of JAM-A. As analyzed by Gotte et al. [25], differences between the two experiments were as follows: (i) the cell density of the latter experiment [25] was much higher, which might facilitate JAM-A dimerization and consequent signal transduction; (ii) Naik et al. [18] used growth factor-depleted matrigel to perform matrigel invasion assays, whereas matrigel used by Gotte et al. [25] contained growth factors. Thus, in the latter experiment, growth factors would activate the JAM-A-mediated signal pathway and promote cell migration. The conflicting outcomes of the two experiments suggest that the activation of the JAM-A-mediated signal pathway might be affected by the microenvironment around the cells. McSherry et al. [21], [23] found that knockdown of JAM-A can reduce MCF-7 cell motility, and this effect was attributed to a lower expression of β1-integrins. However, Murakami et al. [22] observed increased, not decreased migration when JAM-A was blocked by BV11 monoclonal antibody in MMTV-PyVmT-derived tumor cells or 4T1 cell lines, and did not detect a significant change in the level of β1-integrin expression. As analyzed by the Murakami et al. [22], this discrepancy might be attributed to the different cell lines used, and possibly to a different set of integrins expressed. Similarly, the discrepancy in PI3K-Akt-β-catenin signal cascade inhibition by JAM-A between our data and that reported in IECs [27] suggest that inhibition of this signal pathway is cell type-dependent. Meanwhile, as shown by Murakami et al. [22], the lack of JAM-A led to increased cell migration but also sensitivity to apoptosis. Therefore, the final balance of these two aspects of JAM-A functions may determine the overall tumor cell dissemination in vivo (the metastatic ratio tended to be lower in absence of JAM-A although the difference was not significant). JAM-A function is complicated and different functions of JAM-A may be mediated through various signaling molecules, which are expressed differently or activated according to the genetic background of the cells and the microenvironment around the cells, and consequently followed by distinct JAM-A functional responses. The final balance of all these aspects of JAM-A functions may determine overall tumor progression; for example, positively regulate tumor progression in breast cancer [21], [22], [24] and NSCLC, or negatively regulate tumor progression in endometrial carcinoma [19] and pancreatic cancer [20].

In conclusion, our study has demonstrated for the first time that high expression of JAM-A in NSCLC tissues is positively correlated with NSCLC progression. JAM-A can promote NSCLC cell proliferation through cell cycle regulation.
